# Improved production of doubled haploids of winter and spring triticale hybrids via combination of colchicine treatments on anthers and regenerated plants

**DOI:** 10.1007/s13353-016-0387-9

**Published:** 2017-01-06

**Authors:** Aurelia Ślusarkiewicz-Jarzina, Hanna Pudelska, Jolanta Woźna, Tomasz Pniewski

**Affiliations:** Institute of Plant Genetics, Polish Academy of Sciences, Strzeszyńska 34, 60-479 Poznań, Poland

**Keywords:** Anther culture, Doubled haploids, Colchicine treatment, Winter and spring triticale hybrids

## Abstract

Double haploids (DH), obtained during androgenesis in vitro or by genome diploidisation in regenerated haploids, are one type of basic materials used in triticale breeding programmes. The aim of this study was to improve DH production by a combination of colchicine treatment methods on a sample of five winter and five spring triticale hybrids. Colchicine was applied in vitro either in the C17 medium to induce embryo-like structures (ELS) or in the 190-2 medium for green plant (GP) development. Regenerants which remained haploid were immersed in a colchicine solution either when placed on the medium prior to transferring to soil or when growing in pots, followed by the application or absence of cooling. Colchicine treatment during anther culture affected neither ELS nor GP development, but significantly increased the number of DH plants in comparison to spontaneous chromosome doubling. The highest efficiency was recorded when colchicine was applied in the induction medium (55%) versus the regeneration medium (44.5%) or no colchicine treatment (30%). The effectiveness of chromosome duplication in haploid plants ranged from 32 to 64.5% and it was the highest for the treatment on the medium followed by cooling. Individual hybrids differed regarding their capability of regeneration and chromosome doubling, which were consistent only to a low or moderate extent. However, taken together, winter and spring hybrids did not differ significantly. Combined colchicine application resulted in a high yield of DH production, 82.6% for all triticale hybrids, and can provide a considerable number of fertile DH lines for triticale breeding programmes.

**Electronic supplementary material:**

The online version of this article (doi:10.1007/s13353-016-0387-9) contains supplementary material, which is available to authorized users.

## Introduction

Triticale (×*Triticosecale* Wittm.) is one of the most important cereals in Europe and some other regions worldwide. As a synthetic hybrid of wheat (*Triticum*) and rye (*Secale*), it combines valuable traits of both parental species: high feeding value with low growth requirements. Yet, as for other crops, utilisation of triticale depends on breeding programmes, which provide new cultivars with improved utility values or better adapted to the environment conditions. Conventional breeding programmes are relatively time-consuming, as they are based on several rounds of crossing, inbreeding and selection of hybrids. For triticale, this process lasts from 10 to 12 years. As important as favourable economic value is a high degree of plant alignment within a population. Therefore, registration of a new cultivar is preceded by the homozygotisation process, which takes another several years. Variability at the genetic level can be fixed in a short time using the double haploid method to obtain completely homozygous lines from heterozygous initial material. Today, this method is considered an indispensable tool in triticale breeding programmes. However, successful utilisation of doubled haploids depends on the production of large numbers of haploid plants and efficient chromosome doubling.

Triticale haploids (H) and doubled haploids (DH) were usually obtained by androgenesis induced in anther in vitro cultures (González et al. 1997; Tuvesson et al. 2000; Wędzony 2003; Lantos et al. 2014). Androgenesis is a process during which, in place of realisation of a natural development programme, microspores acquire the abilities of cell division, formation of embryo-like structures (ELS) and differentiation processes, leading to plant regeneration. So far, to increase the efficiency of androgenesis induction in anther cultures and then regeneration of green plants (GP) in triticale, various methods were used, such as thermal or nutritional stress prior to planting anthers or modifications of the induction and regeneration media composition, mainly regarding sugars, growth regulators and vitamins (Sharma et al. 1982; Hassawi and Liang 1990; Karsai et al. 1994; Ponitka et al. 1999; Immonen and Robinson 2000; González and Jouve 2000; Ślusarkiewicz-Jarzina et al. 2014). Androgenic plants obtained from anther cultures are mostly haploids, yet DH or aneuploids also occur spontaneously (Muranty et al. 2002; Oleszczuk et al. 2011; Ponitka and Ślusarkiewicz-Jarzina 2011). The number of chromosomes in haploid plants may be doubled by treatment with various compounds, such as oryzalin (Wan et al. 1991), trifluralin (Hansen and Andersen 1998) or caffeine (Thomas et al. 1997), and the most commonly used colchicine (Redha et al. 1998; Arzani and Darvey 2001; Ślusarkiewicz-Jarzina and Ponitka 2003). Chromosome doubling in triticale was most commonly conducted in vivo, by the immersion of regenerated haploid GP in a colchicine solution (Ślusarkiewicz-Jarzina and Ponitka 2003; Würschum et al. 2012). Due to the toxic nature of this agent, and additionally highly concentrated solutions (up to 1000 mg L^−1^) together with the relatively large volumes needed, it was attempted to make the colchicine treatment safer. The main modifications consisted of the in vitro addition of the agent to an ELS-inducing medium during the first stages of androgenesis and applying it at lower concentrations. The focus was on the efficiency of obtaining GP in relation to the number of anthers or isolated microspores plated. Arzani and Darvey (2001) applied the induction medium supplemented with 400 mg L^−1^ colchicine for 72 h. Although GP regeneration decreased, the number of DH plants was several times higher in comparison to the control without colchicine. Similarly, Würschum et al. (2012) tested the addition of colchicine in several combinations of concentration and exposure in cultures of isolated microspores, and despite lowered GP regeneration, the obtained DH exhibited a higher survival rate at comparable fertility to that of the in vivo treatment variant. In turn, in our previous research conducted over many years, good results of DH production were obtained via GP regeneration using a liquid instead of solid C17 medium to induce ELS, followed by in vivo chromosome doubling (Ponitka and Ślusarkiewicz-Jarzina 2007). However, despite enormous progress and development of various methods for the production of triticale DH lines, its potential was not fully realised. Reported methodologies were usually focused on one type of colchicine treatment (e.g. only in vitro to microspores or in vivo on haploids) and targeted to one, i.e. winter or spring, type of triticale.

Here, we present a study that aimed to increase the efficiency of DH production of several winter and spring triticale hybrids by a combined colchicine application. Colchicine treatment was performed either during anther in vitro culture to microspores or ELS, added respectively to the induction or regeneration media, or to regenerated haploid plants, added either prior to or after transfer to soil and followed by different thermal exposure regimes.

## Materials and methods

### Plant materials

The triticale genotypes used in the experiments included five winter and five spring F1 hybrids. Winter hybrids were CT 14259, Mo 35957, Mo 35981, Mo 36082 and Mo 36229, whereas spring hybrids included PJ 486, PJ 525, TJ 15033, TJ 15035 and TJ 15042. All the hybrids were provided by two Polish plant breeding companies: DANKO Plant Breeding Ltd. in Choryń and Plant Breeding Strzelce Ltd. (Supplementary Table 1).

### Colchicine application during in vitro anther cultures

Anther donor plants were grown in the field. Tillers were cut at the stage of uninucleate microspores (after the tetrad stage, but before division into vegetative and generative cells) and cold (4 °C) treated for 6 days in the dark in the mineral salt medium N6 (Chu et al. 1975). Next, spikes were surface-sterilised with 5% calcium hypochlorite for 8 min and subsequently washed several times with sterile distilled water. More than 2000 anthers of each hybrid were isolated and cultured on the C17 medium (Wang and Chen 1983), modified by Ponitka et al. (1999), under previously established conditions (Ślusarkiewicz-Jarzina and Ponitka 2015). Isolated anthers were divided into three pools, corresponding to the variant of colchicine application. According to the first one, anthers were incubated for 24 h in a liquid C17 medium containing colchicine (1.0 mg L^−1^) (Sigma) and then transferred onto the same medium without colchicine, while other anthers were continuously cultured on C17. About 200 anthers were plated per single Ø 60-mm Petri dish and cultured at 28 °C in the dark for 1 month. ELS developed from colchicine-treated anthers were transferred onto the 190-2 medium (Zhuang and Xu 1983) without colchicine (C17_Col_/190-2 variant). ELS from previously untreated anthers were placed for 24 h on the medium 190-2 supplemented with 1.0 mg L^−1^ colchicine following the standard medium (C17/190-2_Col_ variant). Anthers and developed ELS not treated with colchicine at any step were the reference variant (C17/190-2). In each variant, about 30 ELS were collected per Ø 90-mm Petri dish. All regeneration cultures were conducted under fluorescent light at 22 °C with a 16/8 h day/night photoperiod (PAR 80–100 μmol m^−2^ s^−1^, T8 Fluora lamps).

### Ploidy determination and in vivo colchicine treatment of haploid plants

The ploidy level of obtained androgenic GP was determined by flow cytometry of the DNA content in nuclei from leaf cells according to the method described by DeLaat et al. (1987). For ploidy evaluation, a ratio between the average channels of the tissue analysed and that of the 2C peak of a standard was determined using a flow cytometer (PARTEC). Plants containing 1C DNA were identified as haploids, those with 2C DNA as diploids and the remaining plants as aneuploids. Identified DH were grown in a greenhouse, but those of winter genotypes had been first subjected to 8-week vernalisation.

Plants which remained haploid were subjected to different colchicine treatments for 6 h in the light (PAR 200 μmol m^−2^ s^−1^) at 25 °C, all performed using an aqueous solution of the agent (500 mg L^−1^) with a 4% addition of DMSO (dimethyl sulphoxide). In treatments I and II, the plantlets were plated in tubes with the 190-2 medium and immersed in the colchicine solution up to 1 cm above the tillering node, but in treatment II, plants were then additionally cooled. In treatments III and IV, haploids were transplanted to pots with soil and grown for 4 weeks in a growth chamber (25/22 °C day/night, 16/8 h photoperiod, PAR 200 μmol m^−2^ s^−1^). Then, actively tillering plants were removed from the pots, roots were washed out from soil, leaves and roots were trimmed to 3 and 10 cm in length, respectively, and plants were immersed in the colchicine solution, but in treatment IV, plants were then additionally cooled. After any colchicine treatment, plants were washed under running tap water for 24 h. In treatments II and IV, plants after washing were placed in flasks containing tap water replaced daily and cooled for 5 days at 8 °C in 16/8 h light/dark. All plants were finally potted and moved to a greenhouse, where they were grown to maturity.

The effectiveness of chromosome doubling was determined on the basis of fertility of regenerated plants. Fertility percentage was calculated by dividing the number of fertile and partially fertile plants by the total number of plants treated with colchicine.

### Statistical analysis

Experiments were carried out in a randomised design in three replications. The effects of genotype, medium and colchicine application during in vitro culture on regeneration were expressed as mean numbers with standard deviation (SD) of obtained ELS per 100 anthers or GP per 100 ELS or anthers, together with correlations between these parameters. The effectiveness of in vitro colchicine treatment on DH production was calculated as a mean number with SD of DH plants per 100 GP. The effects of genotype and colchicine treatment of haploid plants were expressed as mean numbers with SD of DH obtained per 100 haploids. The results and significance of effects of particular factors were analysed using one-way analysis of variance (ANOVA) and Tukey’s or Fisher’s post-hoc tests at *p* = 0.05, using the Statistica 8.0 statistical package (StatSoft Inc., USA). The total effectiveness of DH production after combined colchicine treatments was calculated as a ratio of obtained DH plants to regenerated GP, for both individual genotypes and winter and spring cultivars jointly.

## Results

Colchicine during in vitro culture was used in three variants, either in the ELS induction medium (C17_Col_/190-2) or in the GP regeneration medium (C17/190-2_Col_) or not used in the control variant (C17/190-2). In total, approximately 10,000 and 13,000 ELS and then 557 and 779 GP were obtained for winter and spring hybrids, respectively. All plants of winter hybrids and randomly selected 533 plants of spring hybrids were analysed by flow cytometry. Plants which remained haploid were treated according to four different regimes. The course and results of in vitro culture, as well as further treatment of obtained plants, are displayed in Supplementary Table 2.

Colchicine applied in the C17 induction medium did not affect ELS development in comparison to untreated anthers, regarding both efficiency and its variation (Table 1). However, marked differences in ELS production were observed between triticale hybrids. Spring hybrids as a whole showed a significantly higher capability to form ELS in comparison to winter hybrids (117 vs. 91 per 100 anthers).

**Table 1:**
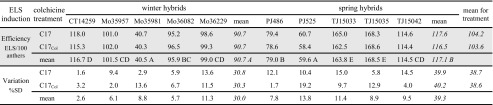
The effect of colchicine treatment of anthers cultured in C17 medium on induction of embryo-like structures (ELS) by triticale hybrids

Efficiency of ELS induction (ELS/100 anthers, mean from three repetitions) and its variation (proportionate ratio of standard deviation to the respective mean) calculated for individual hybrids (*normal font*) and jointly for all winter or spring hybrids or whole ELS induction variants (*italics*). Efficiency analysed using Tukey’s test (*grey shaded block*). The lack of lowercase letter indexes for values in columns indicates insignificant differences between tested ELS induction variants for individual hybrids and winter or spring hybrids considered jointly. Capital indexes in the row with mean values indicate statistically homogenous groups, for individual (*normal font*) and all winter or spring hybrids (*italics*), respectively

Similarly, colchicine treatment had no impact on subsequent GP regeneration. The total regeneration efficiency was nearly identical and with no statistical differences for particular variants of colchicine application and the control. As in the previous step of anther cultures, individual triticale hybrids differed markedly regarding regeneration capability. This time, winter and spring hybrids considered jointly formed plantlets with comparable effectiveness, 5.1 vs. 7.1, when related to 100 anthers as initial explants. However, variation in GP regeneration was greater for spring hybrids (Table 2). For most hybrids, the correlation between ELS and GP development in relation to anthers as initial explants (ELS/100 anthers and GP/100 anthers, respectively) was low or medium (in terms of absolute values, regardless of the negative or positive direction), except for TJ 15033. However, when winter or spring hybrids were considered jointly, the regeneration processes were correlated to a greater extent for the latter ones (Table 2). As ELS or GP development depended on hybrid genotype, the correlation between the two processes was also affected by this factor. Embryogenesis (ELS/100 anthers) and subsequent plantlet regeneration (GP/100 ELS) were inconsistent for all hybrids, except for TJ 15033. A comparison of GP regeneration in relation to ELS (GP/100 ELS) or anthers (GP/100 anthers) indicated medium or low consistency between these parameters for most hybrids. What was important however, was that for individual hybrids, the consistency between ELS and GP formation did not noticeably depend on colchicine treatment (Supplementary Table 3).

**Table 2:**
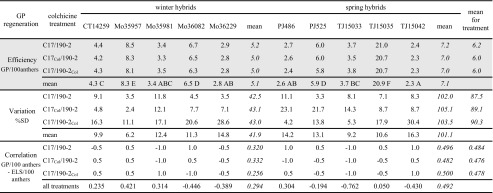
The effect of colchicine treatment of triticale hybrids in medium for ELS induction (C17) or plantlet development (190-2) on resulting efficiency of green plant (GP) regeneration

Regeneration efficiency (GP/100 anthers, mean from three repetitions), its variation (proportionate ratio of standard deviation to the respective mean) and correlation between development of GP/100 anthers and ELS/100 anthers calculated for individual hybrids (*normal font*) and jointly for all winter or spring hybrids or whole variants of colchicine treatment (*italics*). Regeneration efficiency analysed using Tukey’s test (*grey shaded block*). The lack of lowercase letter indexes for values in columns indicates insignificant differences between colchicine treatments for individual genotypes and jointly for winter or spring hybrids. Capital indexes in the row with mean values indicate statistically homogenous groups, for individual hybrids (*normal font*), while a lack of indexes for all winter or spring hybrids (*italics*) indicates insignificant differences between them, respectively

As all plants were regenerated from ELS developed from microspores, it was assumed that the obtained diploids were DH, and not regenerants from diploid tissues of pollen sacs or other anther parts. Flow cytometry analysis of the obtained GP showed that colchicine treatment was significantly more effective when performed at the initial stages of ELS induction (C17_Col_/190-2 variant) instead of the beginning of GP regeneration from fully formed ELS (C17/190-2_Col_), yet both provided significantly higher numbers of DH in comparison to spontaneous diploidisation in the control culture. Also, variation of DH production was the lowest for the C17_Col_/190-2 variant for most of the tested triticale hybrids (Table 3). When colchicine was used in the C17 induction medium, DH accounted for 32.6–83.6% and 46.5–70.6% of obtained GP for winter and spring hybrids, respectively, and 55.3% in total. The addition of colchicine to the 190-2 regeneration medium provided fewer DH, i.e. 28.3–77.0% for winter and 33.8–58.8% for spring hybrids, respectively, and 44.5% in total. The lowest number of DH was recorded for plants obtained under control conditions, without colchicine in both media, i.e. 16.7–67.7% for winter and 18.6–52.9% for spring hybrids, and 29.9% in total. Regardless of the effectiveness of particular variants of in vitro colchicine treatment, individual hybrids varied significantly in terms of the general productivity of DH. Yet, this differed insignificantly for winter and spring hybrids considered jointly, at 42.9 and 43.5%, respectively (Table 3). Apart from DH and haploid plants, some aneuploid plants were also recorded, on average 2.2 and 3.8% for winter and spring hybrids, respectively, mainly in the control culture variant without colchicine (Supplementary Table 2).

**Table 3:**
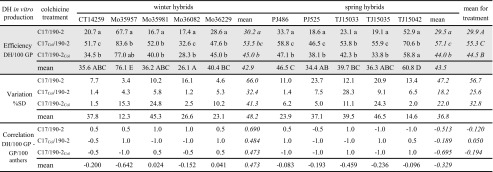
Production of double haploids (DH) by triticale hybrids as the effect of colchicine in vitro treatment in medium for ELS induction (C17) or GP regeneration (190-2)

Efficiency of DH in vitro production (DH/100 GP, means from three repetitions), its variation (proportionate ratio of standard deviation to the respective mean) and correlation between DH/100 GP and development of GP/100 anthers calculated for individual hybrids (*normal font*) or jointly for all winter or spring hybrids and for treatments (*italics*). Efficiency of DH production analysed using Fisher’s test (*grey shaded block*). Lowercase indexes for values in columns indicate significant differences between treatments for individual genotypes (*normal font*) and winter or spring hybrids jointly (*italics*). Capital indexes in the row with mean values or the column with the means for treatments indicate statistically homogenous groups, for individual genotypes (*normal font*) and in vitro colchicine treatments (*italics*), respectively

DH production and regeneration efficiency (measured as GP/100 anthers) were consistent to a medium or low extent. For instance, among hybrid genotypes of remarkable DH production, both genotypes of high and low regeneration capabilities were found, e.g. Mo 35957 and TJ 15042, respectively. An opposite situation was also observed for Mo 36082 or TJ 15035. However, although hybrids differed in DH productivity, this variation seemed lower than in the case of regeneration capability, for both ELS and GP development (Tables 1, 2 and 3). Yet, two trends were revealed after an analysis of correlations between DH production and GP regeneration. Both processes were consistent to a greater extent for winter hybrids, whereas for colchicine treatment, a stronger correlation was observed when it was applied to microspores inside anthers instead of ELS treatment before GP regeneration (Table 3).

Plants of a given hybrid, which remained haploid despite in vitro colchicine treatment or those coming from the control culture, were collected together. Then, they were randomly divided into four equal pools and treated with a colchicine solution according to four different regimes (Supplementary Table 2). Additional DH plants were obtained with an average of 50% efficiency for all five winter hybrids and 44.7% for the five spring hybrids (Table 4). Depending on the hybrid genotype and treatment, the efficiency of chromosome doubling in haploid plants ranged from 14.3 to 78.6%. Comparing the regimes, the effectiveness of the doubling was significantly higher, together with a lower variation when plantlets were colchicine-treated under conditions resembling in vitro culture, i.e. when placed on the 190-2 medium in tubes (regimes I and II), instead of immersion in the colchicine solution of larger plants growing in pots (regimes III and IV). The effectiveness of chromosome doubling was further significantly increased by cooling of the treated plants for 5 days at 8 °C, as in the compared regimes I vs. II and III vs. IV. Finally, significantly, the highest efficiency of chromosome doubling, 64.5% for all hybrids, was observed when plantlets were treated according to regime II, on the medium in tubes and then cooled. In general, the average effectiveness of duplication of the haploid genome was similar for winter and spring hybrids, yet variation of the process was generally lower for winter hybrids (Table 4).

**Table 4:**
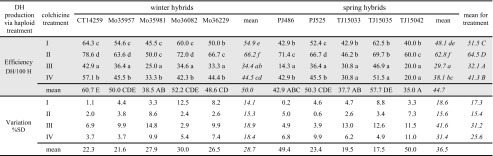
Chromosome doubling in triticale haploid plants remaining after in vitro culture as the effect of different colchicine treatments

Efficiency of chromosome doubling via haploid treatment (DH/100 H, means from three repetitions) and its variation (proportionate ratio of standard deviation to the respective mean) calculated for individual genotypes (*normal font*) and jointly for all winter or spring hybrids or for particular colchicine treatment (*italics*). Efficiency of haploid treatment analysed using Fisher’s test (*grey shaded block*). Lowercase letter indexes for values in columns indicate significant differences between colchicine treatments for individual hybrids (*normal font*) and winter or spring hybrids jointly (*italics*). Capital indexes in the row with mean values or the column with the means for variants of colchicine treatment indicate statistically homogenous groups, for hybrids (*normal font*) and jointly for colchicine treatments (*italics*), respectively

Colchicine treatment: *I* colchicine solution applied on the surface of medium in tube with haploid plant; *II* as in variant I, followed by cooling; *III* regenerated plants growing in vivo immersed in the colchicine solution; *IV* as in variant III, followed by cooling

**Fig. 1:**
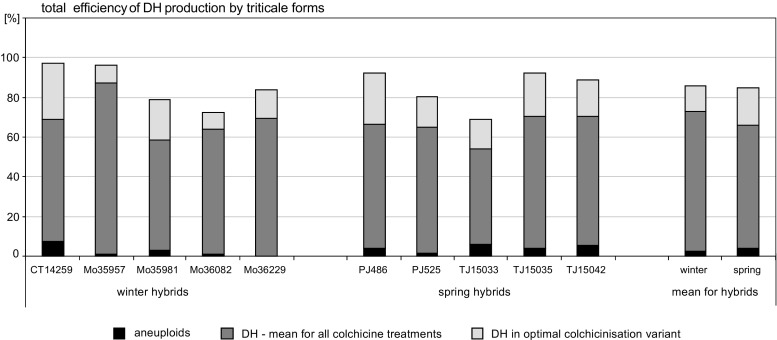
Total efficiency of double haploids (DH) production by triticale hybrids after colchicine treatment during in vitro culture and treatment of developed haploid plants. Mean efficiency of DH production calculated for all variants of in vitro culture and regimes of haploid treatments; the optimal variant comprises the most effective colchicine treatment during in vitro culture (C17_Col_/190-2) and on haploids (regime II: colchicine applied on developed plants on 190-2 medium in tubes, followed by cooling)

A combination of colchicine application during in vitro culture with the following treatment of haploid plants provided efficient production of DH. As the first used, in vitro culture provided a larger proportion of DH plants, but the subsequent treatment of remaining haploid plants substantially increased the final number of obtained DH plants. However, colchicine treatments during in vitro culture and on fully developed haploid plants were comparably effective (Tables 3 and 4). DH production efficiency depended on the hybrid genotype (Fig. 1) and it was usually higher for winter hybrids, on average 72.5 vs. 66% for spring hybrids. However, the optimal variant of colchicine application, i.e. on anthers planted on the C17 medium combined with the treatment of regenerated haploid plants on the 190-2 medium in tubes followed by cooling, resulted not only in greater but also more uniform DH productivity, 83.6 and 81.5% for winter and spring hybrids, respectively (Fig. 1).

The diploid plants were more vigorous in appearance and grew more rapidly than the haploids. Almost all of the doubled haploids obtained after colchicine treatment were fertile, yet most of them only partially, about 40–50%. Among all spikes of a DH plant, a larger proportion had fully filled heads, while some were partially or entirely sterile.

## Discussion

The effect of colchicine treatment on microspores, anthers, pollen callus and ELS as well as obtained haploid plants of wheat or triticale was studied by several authors. It was shown that the effectiveness of obtaining DH varied, depending on genotype and treatment. However, in most such studies, only spring or winter genotypes were used or a limited number of colchicine treatment variants was investigated in a single experiment. Yet, the treatment during in vitro cultures was more often applied than that in vivo on obtained haploids. It was reported that an extended colchicine treatment (1–3 days) of wheat calluses and ELS increased chromosome doubling frequency and was highly reproducible (Ouyang et al. 1994; Mentewab and Sarrafi 1997). More recently, Soriano et al. (2007) applied colchicine during the first hours of culture on anthers or isolated microspores of several spring wheat cultivars and showed that it increased the number of DH lines 3-fold in three genotypes out of four studied. Similarly, Redha and Talaat (2008) noted that, after the use of colchicine, the number of DH plants increased approximately 2-fold, although the number of ELS in two out of three tested spring genotypes decreased 4- or 9-fold. Colchicine in vitro treatment was further developed for triticale. Arzani and Darvey (2001) added colchicine in the MC17 liquid medium at a concentration of 400 mg L^−1^ and showed a 10-fold decrease of GP regeneration efficiency for most studied spring genotypes, yet the number of obtained DH plants was, on average, nine times higher than for spontaneous DH. More recently, Würschum et al. (2012) tested colchicine treatment in several combinations of concentration (0.1, 0.4 and 2.0 mg L^−1^) and duration (24, 48 or 72 h) on a single winter triticale hybrid using the method of isolated microspores cultured on a modified NBP-99 medium (Eudes and Amundsen 2005). In the most effective variant (0.4 mg L^−1^ colchicine for 24 h), GP regeneration increased from 48.3% under control conditions to 72.0%. At the same time, production of fertile DH in relation to ELS also increased from 29.2 to 50.0%, comparably to the results after in vivo colchicine treatment.

In many previous studies of the authors, it was also demonstrated that in vitro and in vivo colchicine treatments could be comparably effective. For example, for seven winter genotypes, the mean proportion of spontaneous DH plants after in vitro culture conducted on C17 and 190-2 media reached 57.5%, while an average efficiency of in vivo chromosome duplication reached 59.6% (Ślusarkiewicz-Jarzina and Ponitka 2003). In another study conducted on 15 winter genotypes, when the regeneration medium was supplemented with kinetin, the efficiency of DH production reached 80.3% of all obtained plants, where spontaneous DH constituted 38%, while the effectiveness of in vivo duplication of the haploid genome reached 70.8% (Ponitka and Ślusarkiewicz-Jarzina 2011). In turn, for six tested spring lines, spontaneous DH accounted for 35.4% after in vitro culture, while DH after in vivo colchicine treatment accounted for 57.1% of all obtained plants (Ślusarkiewicz-Jarzina et al. 2014). A substantial increase in the number of obtained DH plants of six winter and two spring hybrids was recorded when colchicine was applied in the liquid C17 medium, amounting to approximately 3-fold in comparison to the control culture without colchicine (Ślusarkiewicz-Jarzina and Ponitka 2015). It was also found that the use of colchicine at a concentration of minimum 1.0 mg L^−1^ and for a period longer than 24 h decreased the efficiency of ELS and GP production 3-fold.

In this study, different approaches to colchicine treatment were compared and combined to improve the production of DH lines for five winter and five spring hybrids. We tested the previously established and new variants of colchicine treatment in anther culture followed by various treatments of the remaining haploids. Additionally to colchicine application at an early stage of anther culture (microspores before division) in the C17 medium for ELS induction, in vitro treatment of fully developed ELS was tested, during their transfer onto the 190-2 medium for GP regeneration. Fully developed plants, which remained haploid, were further treated according to one of four regimes, i.e. either prior to their transfer to soil when placed individually on the 190-2 medium in tubes, or under in vivo conditions when grown in pots, both followed by the application or absence of cooling.

Individual hybrid genotypes significantly differed in their capability of ELS induction and regeneration, most probably determined by their genetic origin (see Supplementary Table 1). A similar genotype effect was reported many times previously. Apart from that, we noted some regularities in the study. In general, spring hybrids as a whole exhibited a significantly greater capability of ELS development and higher, although insignificant, effectiveness of plantlet regeneration (measured as GP/100 anthers). Also, the correlation between the two processes was higher for spring hybrids taken together. Beyond doubt, the capability of morphogenetic processes in tissue culture remains one of the most multi-faceted issues. However, the results may indicate that androgenesis for triticale could be affected to a larger extent than previously presumed by the general genetic background, which determines the entire developmental programme (here, winter vs. spring). This may be expressed by different response pathways to growth regulators and other culture conditions and, subsequently, their complex effects, similarly to other species (Żur et al. 2015). Moreover, the resulting androgenesis effectiveness may be substantially altered by individual reactions of a genotype to variation-inducing conditions of in vitro culture (Machczyńska et al. 2015).

Regardless of fluctuations in androgenesis observed among individual genotypes, the most important and beneficial feature of colchicine treatment during in vitro culture was that it affected neither ELS formation nor GP regeneration, whereas it significantly increased DH production in comparison to spontaneous chromosome doubling. Colchicine application at the very beginning of anther culture (C17_Col_/190-2 variant) appeared to be more effective and, at the same time, less variable than the treatment of developed ELS (C17/190-2_Col_ variant). This may be explained by the higher susceptibility to colchicine of microspores as separate cells compared to that of multi-cellular and organised ELS. Moreover, colchicine treatment, especially of microspores inside anthers, revealed some additional advantageous effect, as a lower number of aneuploids was produced in comparison to spontaneous chromosome doubling, presumably due to mitosis retardation. Apart from that, it seemed that a genetic factor, crucial for regeneration capability (see above), also plays an essential role in triticale diploidisation. As may be expected, individual hybrids differed significantly regarding DH production, while the mean values for winter and spring hybrids were similar. However, DH production was correlated to a greater extent with GP regeneration for winter hybrids. This trend was more visible when developed plants were exposed on colchicine, both under conditions resembling in vitro culture, where plants were treated after being placed on the medium in tubes, or under in vivo conditions when growing in pots. The efficiency of chromosome doubling was higher and less variable for most individual winter hybrids and, on average, for all of them, although in the latter case, it was insignificant in comparison to spring hybrids. The effectiveness of colchicine treatment in haploid plants was further increased by cooling, especially for winter hybrids. This, again, might indicate, similarly as for androgenetic processes, the significance of the genetic and physiological background in response to agents affecting the developmental programme, especially those directly touching such fundamental processes as cell division.

The presented study confirmed the importance of the time of colchicine application together with its duration and concentration for the production of triticale DH plants. The most effective variant of in vitro colchicine treatment was found to be that in which a low dose of colchicine (1 mg L^−1^ for 24 h) was added to the liquid C17 induction medium at the very early stage of the anther culture. Here, the percentage of DH regenerants reached 55%, in comparison to 44.5% for the ELS treatment or 30% for spontaneous diploidisation. The number of produced DH was further increased by consecutive treatment of haploid plants, up to 64.5%. All in all, the total yield of combined DH production reached 59–87% and 54–70% for winter and spring hybrids, respectively. However, in the optimal variant, it reached 71–95% and 63–88%, on average 83.5 and 81.5%, for winter and spring hybrids, respectively. Thus, although genotype affected DH production at both the stages of in vitro culture and of the haploid plants, a combination of colchicine treatments allowed us, to some extent, to achieve comparable effectiveness of DH production for different hybrids. A majority of obtained DH plants were fully or partially fertile. Therefore, the obtained efficiencies are several times higher than most of those previously reported.

On the basis of the obtained results, we conclude that, to produce a high number of DH lines of triticale hybrids, it is necessary to apply colchicine at two steps: (1) in anther culture, optimally in the medium inducing the androgenesis process and (2) on haploid plants before transfer to soil. The presented study showed the effectiveness of the combined colchicine treatment. The final yield provides a considerable number of fertile DH lines, which may be used in triticale breeding programmes.

## Electronic supplementary material

Below are the links to the electronic supplementary material.

Supplementary Table 1 (DOC 36 kb)

Supplementary Table 2 (DOC 200 kb)

Supplementary Table 3 (DOC 75 kb)
