# Supplementary material for: Improved production of doubled haploids of winter and spring triticale hybrids via combination of colchicine treatments on anthers and regenerated plants
Source: J Appl Genet. 2017 Jan 6;58(3):287–95. doi: 10.1007/s13353-016-0387-9 (PMC5509786; doi:10.1007/s13353-016-0387-9)
Supplement: Supplementary file 1 — (DOC 36 kb) [file 13353_2016_387_MOESM1_ESM.doc]

**Supplementary Table 1.** Origin of tested triticale hybrids. Maternal and paternal forms for crossbreeding, written in that order.

| form | hybrid | crossbreeding | | |
| --- | --- | --- | --- | --- |
| winter | CT14259 | Negoiu | × | Rotondo |
|  | Mo35957 | MAH 32115 | × | TOMKO |
|  | Mo35981 | MAH 33881-14 | × | BOHD 979-1 |
|  | Mo36082 | TOMKO | × | MAH 6912 |
|  | Mo36229 | MAH 33115-4/1 | × | MAH 34615-1 |
| spring | PJ486 | ANDRUS | × | TJ 04068-10 |
|  | PJ525 | TJ 06177-49 | × | MILKARO |
|  | TJ15033 | MAH 33483-5 | × | Mazur |
|  | TJ15035 | MAH 33483-6 | × | Mamut |
|  | TJ15042 | Dublet | × | MAH 33504-9 |
