# Supplementary material for: Improved production of doubled haploids of winter and spring triticale hybrids via combination of colchicine treatments on anthers and regenerated plants
Source: J Appl Genet. 2017 Jan 6;58(3):287–95. doi: 10.1007/s13353-016-0387-9 (PMC5509786; doi:10.1007/s13353-016-0387-9)
Supplement: Supplementary file 2 — (DOC 200 kb) [file 13353_2016_387_MOESM2_ESM.doc]

**Supplementary Table 2.** The course of double haploid production by triticale hybrids using colchicine treatment during *in vitro* culture and treatment of developed haploid plants - number of implanted anthers, obtained embryo-like structures (ELS), green plants (GP), double haploids (DH), aneuploids and remaining haploids (H).

Haploid treatment (grey shaded block) - conducted by treatment with colchicine solution (500 mg l^-1^ for 6 h) of developed plants which remained haploid after all three *in vitro* culture variants. Haploids were collected together and then randomly divided into four equal pools (±1) for four different treatments: I - plants placed separately on 190-2 medium in tubes; II - as in variant I, followed by cooling at 8°C for 5 days; III - plants growing *in vivo* (in pots); IV - as in variant III, followed by cooling at 8°C for 5 days. Number of DH and H plants refers to all treated plants of a given hybrid or whole winter or spring groups.

| Triticale  hybrid | | *In vitro*  culture | anthers | ELS | GP | cytometry  analysed | DHs after  *in vitro* | aneuploids | haploids for  treatment | plants after colchicine treatment | | | | | |
| --- | --- | --- | --- | --- | --- | --- | --- | --- | --- | --- | --- | --- | --- | --- | --- |
|  |  |  |  |  |  |  |  |  |  | ploidy | I | II | III | IV | total |
| winter | CT  14259 | C17/190-2 | 800 | 930 | 35 | 35 | 6 | 6 | 23 | DH | 9 | 11 | 6 | 8 | 34 |
|  |  | C17_Col_/190-2 | 700 | 807 | 29 | 29 | 15 | 0 | 14 |  |  |  |  |  |  |
|  |  | C17/190-2_Col_ | 700 | 840 | 30 | 30 | 10 | 1 | 19 | H | 5 | 3 | 8 | 6 | 22 |
|  |  | total | 2200 | 2577 | 94 | 94 | 31 | 7 | 56 |  |  |  |  |  |  |
|  | Mo  35957 | C17/190-2 | 750 | 750 | 64 | 64 | 42 | 2 | 20 | DH | 6 | 7 | 4 | 5 | 22 |
|  |  | C17_Col_/190-2 | 750 | 765 | 62 | 62 | 52 | 0 | 10 |  |  |  |  |  |  |
|  |  | C17/190-2_Col_ | 750 | 765 | 61 | 61 | 47 | 0 | 14 | H | 5 | 4 | 7 | 6 | 22 |
|  |  | total | 2250 | 2280 | 187 | 187 | 141 | 2 | 44 |  |  |  |  |  |  |
|  | Mo  35981 | C17/190-2 | 700 | 285 | 24 | 24 | 3 | 1 | 20 | DH | 5 | 6 | 3 | 4 | 18 |
|  |  | C17_Col_/190-2 | 770 | 310 | 25 | 25 | 13 | 0 | 12 |  |  |  |  |  |  |
|  |  | C17/190-2_Col_ | 750 | 305 | 26 | 26 | 10 | 1 | 15 | H | 6 | 6 | 9 | 8 | 29 |
|  |  | total | 2220 | 900 | 75 | 75 | 26 | 2 | 47 |  |  |  |  |  |  |
|  | Mo  36082 | C17/190-2 | 750 | 714 | 47 | 47 | 8 | 1 | 38 | DH | 15 | 18 | 9 | 11 | 53 |
|  |  | C17_Col_/190-2 | 650 | 627 | 46 | 46 | 15 | 0 | 31 |  |  |  |  |  |  |
|  |  | C17/190-2_Col_ | 750 | 714 | 46 | 46 | 13 | 0 | 33 | H | 10 | 7 | 17 | 15 | 49 |
|  |  | total | 2150 | 2055 | 139 | 139 | 36 | 1 | 102 |  |  |  |  |  |  |
|  | Mo  36229 | C17/190-2 | 700 | 690 | 21 | 21 | 6 | 0 | 15 | DH | 5 | 6 | 3 | 4 | 18 |
|  |  | C17_Col_/190-2 | 750 | 745 | 21 | 21 | 10 | 0 | 11 |  |  |  |  |  |  |
|  |  | C17/190-2_Col_ | 750 | 740 | 20 | 20 | 9 | 0 | 11 | H | 5 | 3 | 6 | 5 | 19 |
|  |  | total | 2200 | 2175 | 62 | 62 | 25 | 0 | 37 |  |  |  |  |  |  |
|  | all  forms | C17/190-2 | 3700 | 3369 | 191 | 191 | 65 | 10 | 116 | DH | 40 | 48 | 25 | 32 | 145 |
|  |  | C17_Col_/190-2 | 3620 | 3254 | 183 | 183 | 105 | 0 | 78 |  |  |  |  |  |  |
|  |  | C17/190-2_Col_ | 3700 | 3364 | 183 | 183 | 89 | 2 | 92 | H | 31 | 23 | 47 | 40 | 141 |
|  |  | total | 11020 | 9987 | 557 | 557 | 259 | 12 | 286 |  |  |  |  |  |  |
| spring | PJ  486 | C17/190-2 | 700 | 556 | 20 | 20 | 6 | 2 | 12 | DH | 3 | 5 | 1 | 3 | 12 |
|  |  | C17_Col_/190-2 | 700 | 550 | 18 | 17 | 10 | 0 | 7 |  |  |  |  |  |  |
|  |  | C17/190-2_Col_ | 700 | 556 | 17 | 17 | 8 | 0 | 9 | H | 4 | 2 | 6 | 4 | 16 |
|  |  | total | 2100 | 1662 | 55 | 54 | 24 | 2 | 28 |  |  |  |  |  |  |
|  | PJ  525 | C17/190-2 | 700 | 425 | 45 | 45 | 8 | 0 | 37 | DH | 11 | 14 | 8 | 10 | 43 |
|  |  | C17_Col_/190-2 | 700 | 409 | 43 | 42 | 19 | 0 | 23 |  |  |  |  |  |  |
|  |  | C17/190-2_Col_ | 800 | 485 | 44 | 43 | 15 | 2 | 26 | H | 10 | 7 | 14 | 12 | 43 |
|  |  | total | 2200 | 1319 | 132 | 130 | 42 | 2 | 86 |  |  |  |  |  |  |
|  | TJ  15033 | C17/190-2 | 750 | 1237 | 28 | 28 | 6 | 2 | 20 | DH | 6 | 6 | 4 | 4 | 20 |
|  |  | C17_Col_/190-2 | 800 | 1300 | 28 | 27 | 11 | 1 | 15 |  |  |  |  |  |  |
|  |  | C17/190-2_Col_ | 720 | 1180 | 27 | 28 | 8 | 2 | 18 | H | 8 | 7 | 9 | 9 | 33 |
|  |  | total | 2270 | 3717 | 83 | 83 | 25 | 5 | 53 |  |  |  |  |  |  |
|  | TJ  15035 | C17/190-2 | 800 | 1337 | 165 | 72 | 13 | 4 | 55 | DH | 20 | 23 | 15 | 17 | 75 |
|  |  | C17_Col_/190-2 | 700 | 1180 | 145 | 69 | 38 | 1 | 30 |  |  |  |  |  |  |
|  |  | C17/190-2_Col_ | 700 | 1188 | 145 | 71 | 23 | 3 | 45 | H | 12 | 10 | 17 | 16 | 55 |
|  |  | total | 2200 | 3705 | 455 | 212 | 74 | 8 | 130 |  |  |  |  |  |  |
|  | TJ  15042 | C17/190-2 | 750 | 850 | 18 | 18 | 9 | 1 | 8 | DH | 2 | 3 | 1 | 1 | 7 |
|  |  | C17_Col_/190-2 | 800 | 915 | 18 | 18 | 12 | 1 | 5 |  |  |  |  |  |  |
|  |  | C17/190-2_Col_ | 770 | 892 | 18 | 18 | 10 | 1 | 7 | H | 3 | 2 | 4 | 4 | 13 |
|  |  | total | 2320 | 2657 | 54 | 54 | 31 | 3 | 20 |  |  |  |  |  |  |
|  | all  forms | C17/190-2 | 3700 | 4405 | 276 | 183 | 42 | 9 | 132 | DH | 42 | 51 | 29 | 35 | 157 |
|  |  | C17_Col_/190-2 | 3700 | 4354 | 252 | 173 | 90 | 3 | 80 |  |  |  |  |  |  |
|  |  | C17/190-2_Col_ | 3690 | 4301 | 251 | 177 | 64 | 8 | 105 | H | 37 | 28 | 50 | 45 | 160 |
|  |  | total | 11090 | 13060 | 779 | 533 | 196 | 20 | 317 |  |  |  |  |  |  |
| all triticale hybrids | | C17/190-2 | 7400 | 7774 | 467 | 374 | 107 | 19 | 248 | DH | 82 | 99 | 54 | 67 | 302 |
|  |  | C17_Col_/190-2 | 7320 | 7608 | 435 | 356 | 195 | 3 | 158 |  |  |  |  |  |  |
|  |  | C17/190-2_Col_ | 7390 | 7665 | 434 | 360 | 153 | 10 | 197 | H | 68 | 51 | 97 | 85 | 301 |
|  |  | total | 22110 | 23047 | 1336 | 1090 | 455 | 32 | 603 |  |  |  |  |  |  |
