# Supplementary material for: Improved production of doubled haploids of winter and spring triticale hybrids via combination of colchicine treatments on anthers and regenerated plants
Source: J Appl Genet. 2017 Jan 6;58(3):287–95. doi: 10.1007/s13353-016-0387-9 (PMC5509786; doi:10.1007/s13353-016-0387-9)
Supplement: Supplementary file 3 — (DOC 75 kb) [file 13353_2016_387_MOESM3_ESM.doc]

**Supplementary Table 3.** The effect of colchicine treatment of triticale hybrids in medium for ELS induction (C17) or plantlet regeneration (190-2) on GP development directly from ELS.

Regeneration efficiency (GP/100 ELS, mean from 3 repetitions), its variation (proportionate ratio of standard deviation to the respective mean) and correlations between development of GP/100 ELS and ELS/100 anthers and between GP/100 anthers and GP/100 ELS calculated for individual hybrids (normal font) and jointly for all winter or spring hybrids or whole variants of colchicine treatment (italic). Regeneration efficiency analysed using Tukey’s test (grey shaded block). The lack of small letter indexes for values in columns indicates insignificant differences between colchicine treatments for individual genotypes and jointly for winter or spring hybrids. Capital indexes in the row with mean values indicate statistically homogenous groups for individual hybrids (normal font), while a lack of indexes for all winter or spring hybrids (italic) indicates insignificant differences between them, respectively.

| GP regeneration | colchicine treatment | winter hybrids | | | | | | spring hybrids | | | | | | mean  for  treatment |
| --- | --- | --- | --- | --- | --- | --- | --- | --- | --- | --- | --- | --- | --- | --- |
|  |  | CT14259 | Mo35957 | Mo35981 | Mo36082 | Mo36229 | mean | PJ486 | PJ525 | TJ15033 | TJ15035 | TJ15042 | mean |  |
| Efficiency  GP/100 ELS | C17/190-2 | 3.8 | 8.5 | 8.4 | 7.0 | 2.9 | *6.1* | 3.4 | 9.9 | 2.3 | 12.6 | 2.1 | *6.1* | *6.1* |
|  | C17_Col_/190-2 | 3.4 | 8.1 | 8.1 | 6.7 | 2.8 | *5.8* | 3.3 | 10.3 | 2.2 | 12.3 | 2.0 | *6.0* | *5.9* |
|  | C17/190-2_Col_ | 3.6 | 8.0 | 8.5 | 6.6 | 2.8 | *5.9* | 3.1 | 9.5 | 2.3 | 12.2 | 2.0 | *5.8* | *5.9* |
|  | mean | 3.6 B | 8.2 D | 8.3 D | 6.8 C | 2.8 AB | *5.9* | 3.3 AB | 9.9 E | 2.3 A | 12.4 E | 2.0 A | *6.0* |  |
| Variation  %SD | C17/190-2 | 5.3 | 1.2 | 4.8 | 10.0 | 13.8 | *40.0* | 17.6 | 9.1 | 4.3 | 17.5 | 0.0 | *75.8* | *59.4* |
|  | C17_Col_/190-2 | 17.6 | 11.1 | 3.7 | 10.4 | 3.6 | *41.2* | 21.2 | 23.3 | 4.5 | 10.6 | 10.0 | *77.4* | *61.6* |
|  | C17/190-2_Col_ | 16.7 | 12.5 | 1.2 | 1.5 | 10.7 | *41.2* | 3.2 | 5.3 | 17.4 | 23.0 | 5.0 | *77.2* | *60.5* |
|  | mean | 13.0 | 8.7 | 3.7 | 7.8 | 9.2 | *40.0* | 14.8 | 13.6 | 9.6 | 15.4 | 6.0 | *75.0* |  |
| Correlation  GP/100 ELS -  ELS/100 anthers | C17/190-2 | 1.0 | 0.5 | -0.5 | 0.5 | -0.5 | *-0.547* | -0.5 | 0.5 | 0.5 | -0.5 | n/a | *0.042* | *-0.097* |
|  | C17_Col_/190-2 | -0.5 | 1.0 | -0.5 | 0.5 | -1.0 | *-0.544* | 0.5 | -0.5 | 1.0 | 0.5 | 0.5 | *0.016* | *-0.099* |
|  | C17/190-2_Col_ | -0.5 | -0.5 | -0.5 | -0.5 | 0.5 | *-0.616* | 0.5 | 0.5 | 1.0 | 0.5 | -1.0 | *0.080* | *-0.088* |
|  | all treatments | -0.098 | -0.135 | -0.206 | -0.005 | -0.161 | *-0.551* | -0.151 | -0.266 | 0.773 | 0.174 | -0.203 | *0.045* |  |
| Correlation  GP/100 ELS -  GP/100 anthers | C17/190-2 | -0.5 | 1.0 | 0.5 | 0.5 | -0.5 | *0.574* | -0.5 | -0.5 | -0.5 | 0.5 | n/a | *0.835* | *0.768* |
|  | C17_Col_/190-2 | -1.0 | 0.5 | 0.5 | -0.5 | 0.5 | *0.551* | -0.5 | -0.5 | -1.0 | -1.0 | 1.0 | *0.781* | *0.728* |
|  | C17/190-2_Col_ | 0.5 | 0.5 | -0.5 | 0.5 | -1.0 | *0.503* | 0.5 | -0.5 | -1.0 | -0.5 | 1.0 | *0.786* | *0.715* |
|  | all treatments | 0.138 | 0.491 | 0.323 | 0.074 | -0.552 | *0.544* | -0.321 | -0.413 | -0.410 | -0.345 | 0.643 | *0.800* |  |
